# Physician-Suggested Innovative Methods for Health System Resilience amidst Workforce Emigration and Sociopolitical Unrest in Nigeria: A Survey-Based Study

**DOI:** 10.5334/aogh.4025

**Published:** 2023-02-16

**Authors:** Tega Ebeye, HaEun Lee, Abi Sriharan

**Affiliations:** 1 University of Toronto, Temerty Faculty of Medicine, Toronto, Canada; 2 University of Toronto, Institute of Health Policy, Management and Evaluation, Toronto, Canada; 3 University of Michigan, Center for Global Health Equity, Ann Arbor, Michigan, USA

**Keywords:** resilience, emigration, brain drain, human resources for health, sociopolitical unrest

## Abstract

**Introduction:**

Physician emigration (the *brain drain*) and sociopolitical unrest significantly contribute to the instability of many low- and middle-income countries’ healthcare systems. However, limited literature captures the *locally driven* and *context specific* suggestions to promote and sustain these health systems’ resilience. Thus, the purpose of this study is to 1) understand the effects of physician emigration and sociopolitical unrest on Nigeria’s healthcare system, and to 2) synthesize solutions suggested by Nigeria-trained physicians in the form of a resilience framework.

**Methods:**

An anonymous online survey was conducted among Nigeria-trained physicians. Respondents were recruited using convenience and snowball sampling methods via a *WhatsApp* group for Nigeria-trained doctors. Quantitative data were analyzed using *Stata 17* and qualitative themes were coded by two independent researchers.

**Results:**

The final sample included 49 Nigeria-trained physicians—35 physicians practicing in Nigeria and 14 emigrated physicians. All of the physicians currently practicing in Nigeria have considered emigrating, with 79% of them having concrete plans to emigrate in the next five years. Among emigrated physicians, factors such as remuneration (92%) and socioeconomic state of the country (92%) contributed to their decision to emigrate. Suggestions to enhance health system resilience fell into six broad themes: 1) policy and politics, 2) funding and resources, 3) organization and structure, 4) training and education, 5) research and primary health, and 6) health for peace initiatives.

**Conclusions:**

The healthcare system is currently unstable and vulnerable due to physician emigration and sociopolitical unrest. To develop and implement solutions to mitigate these issues, capturing the locally trained physicians’ perspectives are critical. While each country’s healthcare system is unique, countries with similar strains can adapt this model for resilience building. Future studies should focus on adapting the model in different countries with policy-level action points.

## Background

Healthcare systems play pivotal roles in maintaining and promoting the health of populations, while also contributing to the economy, structure, and development of nations. Thus, the instability of healthcare systems is a global health dilemma with implications that undermine the survival and sustainability of these systems worldwide. Hence, health system resilience has emerged as a prominent topic in global health discourse; with emphasis on the fact that resilience is not an action to be implemented but rather a dynamic process of investments and reforms [1,2,3,4].

The diversity of factors that destabilize health systems—including economic crises, climate change, natural disasters, disease outbreaks, migration, conflict, and evolving population—points to the exploration of reasons that leave some healthcare systems more susceptible to instability than others [1]. The emigration of physicians and other healthcare workers from low- and middle-income countries (LMICs) to high-income countries (HICs), colloquially referred to as the *brain drain*, is a factor that has been a prominent discussion in global health spheres for years—presenting a complex set of decisions and relationships that affect the development of international healthcare systems [5]. Between 2005 and 2015, there was over a 70% increase in Africa-trained doctors who subsequently entered the US workforce with a continual and steady increase over the last half-century [6,7]. Thus, this emigration creates imbalances and gaps in the LMICs healthcare systems’ workforce—some of which are already struggling with the other factors affecting the stability of health systems. The World Health Organization (WHO) cites workforce as a necessary building block for health systems; yet estimates a projected shortfall of 15 million healthcare workers by 2030, mostly in LMICs who have an average physician density of 17 per 100,000 population as compared to an average of 300 per 100,000 in HICs [8,9,10].

Given that sub-Saharan Africa serves as a colossal source region of physicians emigrating to HICs, the WHO estimates that one in four doctors will leave Africa to pursue jobs abroad [11]. Specifically, of the international medical graduates from sub-Saharan African countries practicing in the US, 44.5% are from Nigeria [7]. Studies have listed unrest, safety, and security concerns as major *push factors* for the move to HICs, emphasizing that LMICs with broad-based unrest are more susceptible to the factors affecting the stability of health systems [12,13]. Furthermore, increasing unrest and national security concerns in Nigeria in the form of the *Boko Haram* insurgency, may further drive health workforce emigration [14]. Despite the crippling impact of workforce emigration on LMICs health systems, there are limited studies consulting physicians from LMICs—a seemingly untapped resource—for solution generation. Hence, this study aims to understand the effects of workforce emigration and sociopolitical unrest on the Nigerian healthcare system and to develop an innovative framework for resilience based on physician’s suggestions.

## Methods

This study used a mixed methods approach with a cross-sectional anonymous online survey targeting Nigeria-trained physicians. Respondents were recruited using convenience and snowball sampling methods in which the primary group of respondents were solicited via *WhatsApp*, an internationally available messenger platform widely used in Africa. The link to the survey was initially distributed through a *WhatsApp* group for Nigeria-trained doctors containing 259 Nigeria-trained physicians practicing in the United Kingdom. The group members were encouraged to distribute the survey link to other colleagues, with other Nigeria-trained physicians not in the group solicited via snowball sampling. The inclusion criteria included having attended and graduated from a Nigerian medical school, with the assumption that this target sample would have the most insight on resilience building—with regards to emigration and unrest as destabilizing factors—based on being ingrained in the system right from training. Due to the anonymous nature of the survey and the ability of individuals to forward the link, the total denominator for potential respondents, as well as the response rate, cannot be determined. Prior to the dissemination of the survey, ethics approval was sought from the Research Ethics Board (REB), and approved by the REB of the primary author’s institution.

### Survey Instrument

The survey consisted of three main sections. The first section included demographic questions such as age, gender, place of birth, and medical school. The second section had two separate versions. The first version targeted Nigeria-trained physicians practicing in Nigeria at the moment of data collection. It included both open and closed ended questions regarding job satisfaction, emigration plans, the effects of physician emigration on the Nigerian healthcare system, and reforms the Nigerian healthcare system can adapt to remain resilient in the face of physician emigration and socio-political unrest. The second version targeted Nigeria-trained physicians practicing in countries outside of Nigeria at the moment of data collection. It included both open and closed ended questions regarding current country of practice, factors leading to emigration, job satisfaction while in Nigeria, and job satisfaction in the current country of practice.

Data were collected using the online survey software *Qualtrics*, allowing respondents to complete the survey at a time and location of their convenience. The survey introduction asked for respondents’ consent, informing them that the survey was voluntary and that the data will remain anonymous. Additionally, the survey included a trigger warning mentioning that the questions about emigration and working in areas of unrest may generate discomfort, and the respondents may pause or discontinue the survey at any moment. The survey link was available for two months, from May 1, 2022, until June 30, 2022. All data were automatically saved in *Qualtrics* and downloaded into Microsoft Excel upon completion.

### Data Analysis

Quantitative data were cleaned and analyzed using *Stata 17* (StataCorp, College Station, TX, USA) and qualitative data were organized and analyzed by two independent researchers using Microsoft Word. Quantitative data included respondent demographics and other closed ended questions such as a Likert scale for job satisfaction and factors influencing emigration. Qualitative data included open-ended questions such as how Nigeria’s healthcare system has been affected by unrest, suggestions on how Nigeria’s healthcare system can remain resilient in the face of unrest, and means to improve physician retention rate.

The qualitative data were examined by two independent researchers who independently identified the preliminary themes. A codebook was developed and the two researchers independently hand-coded the data. The coded data were then compared to identify all the discrepancies discussed until the two researchers reached a consensus regarding the appropriate code. If no appropriate code was found, additional codes were developed with the consensus of both researchers. The codebook and original themes were then revisited and discussed until the overarching themes emerged.

## Results

**Figure 1:**
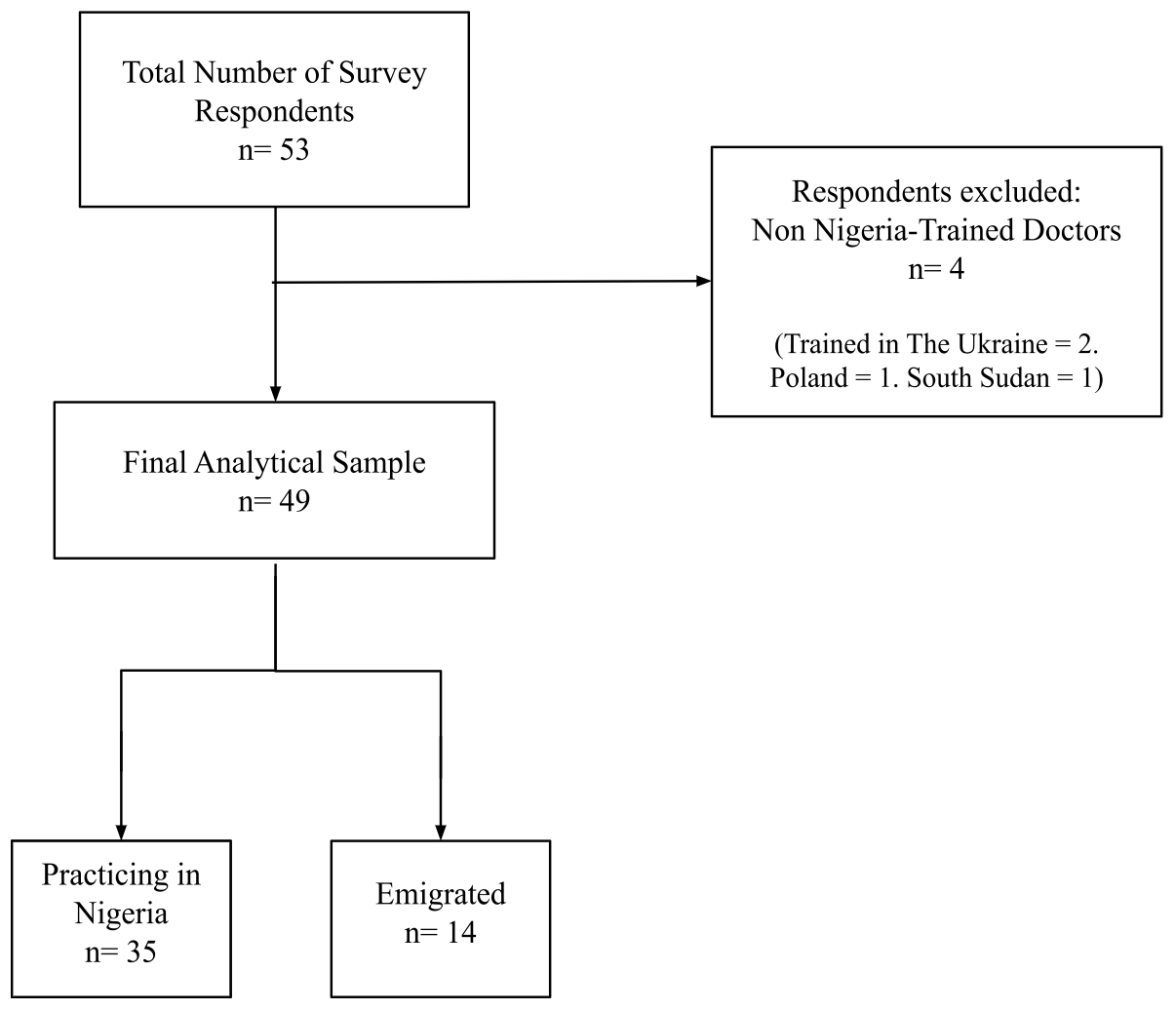
Flowchart of participating physicians.

Figure 1 presents the flowchart of participating physicians. A total of 53 physicians participated in the survey. Four were excluded per exclusion criteria. The final analytic sample of 49 participants included 35 physicians practicing in Nigeria and 14 practicing outside of Nigeria at the time of survey.

### Demographics

The demographic characteristics of the total sample—stratified by location of current practice—are presented in Table 1.

**Table 1:** Demographics of participants.


CHARACTERISTICS	N (%)	PHYSICIANS PRACTICING IN NIGERIA	PHYSICIANS PRACTICING OUTSIDE OF NIGERIA

Total	49 (100.00)	35 (71.43)	14 (28.57)

Age^a^			

29 or younger	19 (38.78)	17 (48.57)	2 (14.29)

30–39	26 (53.06)	16 (45.71)	10 (71.43)

40–49	4 (8.16)	2 (5.71)	2 (14.29)

Gender^b^			

Female	22 (44.90)	13 (37.14)	9 (64.29)

Male	27 (55.10)	22 (62.86)	5 (35.71)

Were you born in Nigeria?			

Yes	48 (97.96)	35 (100.00)	13 (92.86)

No	1 (2.04)	–	1 (7.14)

Where did you attend high school?			

Nigeria	48 (97.96)	34 (97.14)	14 (100.00)

Other	1 (2.04)	1 (2.86)	–

Where is the medical school you graduated from located?			

Nigeria	49 (100.00)	35 (100.00)	14 (100.00)

Other	–	–	–

Location of medical school (city, state)			

Abuja	3 (6.12)	3 (8.57)	–

Benin City, Edo	2 (4.08)	–	2 (14.29)

Calabar, Cross River	1 (2.04)	–	1 (7.14)

Elele, Rivers	1 (2.04)	1 (2.86)	–

Ibadan, Oyo	4 (8.16)	1 (2.86)	3 (21.43)

Ilorin, Kwara	2 (4.08)	–	2 (14.29)

Iwo, Osun	4 (8.16)	4 (11.43)	–

Jos, Plateau	19 (38.78)	19 (54.29)	–

Lagos	3 (6.12)	2 (5.71)	1 (7.14)

Maiduguri, Borno	8 (16.33)	5 (14.29)	3 (21.43)

Port Harcourt, Rivers	2 (4.08)	–	2 (14.29)

In what year did you graduate from medical school?			

2010 or before	5 (10.20)	2 (5.71)	3 (21.43)

2011–2015	20 (40.82)	10 (28.57)	10 (71.43)

2016–2020	18 (36.73)	17 (48.57)	1 (7.14)

2021 or after	6 (12.24)	6 (17.14)	–

Did you undergo specialty training (residency training)?			

No	37 (75.51)	27 (77.14)	10 (71.43)

Yes	12 (24.49)	8 (22.86)	4 (28.57)

If yes, in which country?			

Nigeria	11 (91.67)	8 (100.00)	3 (75.00)

UK	1 (8.33)	–	1 (25.00)

What is your current medical specialty?			

Family/general practice	18 (36.73)	12 (34.29)	6 (42.86)

Internal medicine, (e.g., cardiology, respirology, gastroenterology)	3 (6.12)	–	3 (21.43)

Surgery (e.g., plastic surgery, orthopedic surgery)	–	–	–

Pediatrics	–	–	–

Psychiatry	3 (6.12)		3 (21.43)

Obstetrics/gynecology	2 (4.08)	2 (5.71)	–

Urology	–	–	–

Unspecialized/Medical Officer	17 (34.69)	16 (45.71)	1 (7.14)

Other	6 (12.24)	5 (14.29)	1 (7.14)


^a^: Categories for ages 50–59 and 60 or older presented but had no observations. ^b^: Options for “non-binary” and “prefer not to answer” provided but had no observations. All variable percentages were calculated with missing observations so the total may not add up to 100.00.

### Job safety, satisfaction, emigration intent, and reasons for emigration

The job safety, satisfaction, and emigration intent of physicians currently practicing in Nigeria are presented in Table 2. Regarding overall satisfaction of the current practice, 41% of the physicians currently practicing in Nigeria are dissatisfied to some degree with only one participant stating that they were “extremely satisfied” with their current practice. In addition, half of them mentioned feeling unsafe at work. When asked about emigration plans, all of the physicians currently practicing in Nigeria answered that they have considered emigrating, from 41% answering that they are “always” considering practicing abroad, to only one participant answering that they “rarely” do. Majority of the participants (79%) had concrete plans to emigrate from Nigeria in the next five years. Finally, when asked about confidence in their current practice to withstand the surrounding instability in the long run, close to half of the participants (47%) answered “not confident at all.”

**Table 2:** Job safety, satisfaction, and emigration intent of physicians practicing in Nigeria.


QUESTION	N (%)

Total	35 (100)

What state in Nigeria do you currently practice?	

Abuja	5 (14.29)

Adamawa	3 (8.57)

Akwa Ibom	1 (2.86)

Bauchi	1 (2.86)

Benue	1 (2.86)

Cross River	1 (2.86)

Delta	1 (2.86)

Kaduna	2 (5.71)

Katsina	1 (2.86)

Lagos	4 (11.43)

Nasarawa	1 (2.86)

Niger	1 (2.86)

Plateau	12 (34.29)

Rivers	1 (2.86)

How satisfied are you with the overall functioning of your practice?	

Extremely Satisfied	1 (2.94)

Somewhat Satisfied	14 (41.18)

Neither satisfied nor Dissatisfied	5 (14.71)

Somewhat Dissatisfied	10 (29.41)

Extremely Dissatisfied	4 (11.76)

Do you ever consider emigrating from Nigeria to practice abroad?	

Always	14 (41.18)

Often	8 (23.53)

Sometimes	11 (32.35)

Rarely	1 (2.94)

Never	–

Do you have plans to emigrate from Nigeria in the next 5 years?	

Yes	27 (79.41)

No	7 (20.59)

Do you feel safe in your place of work?	

Yes	17 (50.00)

No	17 (50.00)

How confident are you that your current practice can withstand the surrounding instability in the long run?	

Completely Confident	2 (5.88)

Fairly Confident	3 (8.82)

Somewhat Confident	8 (23.53)

Slightly Confident	5 (14.71)

Not Confident at all	16 (47.06)


All variable percentages were calculated with missing observations so the total may not add up to 100.00.

Emigrated physicians’ country of practice, previous practices in Nigeria, past and current job satisfaction, and reasons for emigration are presented in Table 3. Eight of the fourteen emigrated physicians were practicing in the United Kingdom (57%), three in the United States (21%), and three in Canada (21%). All of them had practiced medicine in Nigeria before emigration with eight (57%) having practiced in more than one state in Nigeria. More than half of them practiced for between five to ten years in Nigeria before emigrating. When asked about the overall satisfaction regarding their practice in Nigeria, six (43%) answered somewhat dissatisfied and five (36%) answered extremely dissatisfied. All except for two emigrated from Nigeria in the past five years and the majority of them (93%) were either extremely satisfied or somewhat satisfied with their current practice outside of Nigeria. Of the list of factors that led to emigration, physician remuneration (93%), socioeconomic state of the country (93%), quality of facilities (86%), and unrest (79%) were most frequently identified.

**Table 3:** Practices, job satisfaction, and reasons for emigration for emigrated physicians.


QUESTION	N (%)

Total	14 (100.00)

Where are you currently practicing?	

Canada	3 (21.43)

United Kingdom	8 (57.14)

United States	3 (21.43)

Have you ever practiced medicine in Nigeria?	

No	–

Yes	14 (100.00)

If yes, where/which state?	

Abuja	2 (14.29)

Bayelsa, Rivers, Anambra	1 (7.14)

Benue, Abuja	1 (7.14)

Edo	1 (7.14)

Edo, Kaduna	1 (7.14)

Lagos	1 (7.14)

Lagos, Bauchi	1 (7.14)

Lagos, Kwara	1 (7.14)

Osun, Ekiti, Kwara, Ondo	1 (7.14)

Oyo, Adamawa, Osun	1 (7.14)

Plateau	3 (20.00)

Plateau, Ekiti, Abuja	1 (7.14)

How many years did you practice in Nigeria?	

< 1 year	–

1 to 2 years	3 (21.43)

2 to 5 years	2 (14.29)

5 to 10 years	8 (57.14)

More than 10 years	1 (7.14)

How satisfied were you with the overall functioning of your practice in Nigeria?	

Extremely Satisfied	–

Somewhat Satisfied	2 (14.29)

Neither satisfied nor Dissatisfied	1 (7.14)

Somewhat Dissatisfied	6 (42.86)

Extremely Dissatisfied	5 (35.71)

Which (if any) of the following factors led you to emigrate from Nigeria? Please select all that apply:	

Physician remuneration (pay)	13 (92.85)

Quality of facilities	12 (85.71)

Unrest (sociopolitical, civil and other broad–based unrest)	11 (78.57)

Personal safety	7 (50.00)

Socioeconomic state of the country	13 (92.85)

Quantity and availability of allied health disciplines (e.g., nursing, physical therapy, etc.)	2 (14.28)

Shortcomings in governance and health services management	10 (71.42)

Opportunity for continuing education (residency training, etc.)	9 (64.28)

Workload	2 (14.28)

Other	4 (28.57)

Was the functioning of the health system you practiced in Nigeria affected by unrest (e.g., crises, Boko Haram terrorism, kidnappings, crime etc.)?	

No	6 (42.86)

Yes	8 (57.14)

In what year did you arrive in your current country of practice?	

Prior to 2017	2 (14.29)

2017	3 (21.43)

2018	2 (14.29)

2019	2 (14.29)

2020	4 (28.57)

Is your current specialty the same as the one in which you trained and/or practiced before emigrating?	

No	7 (50.00)

Yes	7 (50.00)

How satisfied are you with the overall functioning of your current practice?	

Extremely Satisfied	4 (28.57)

Somewhat Satisfied	9 (64.29)

Neither satisfied nor Dissatisfied	1 (7.14)

Somewhat Dissatisfied	–

Extremely Dissatisfied	–


*All variable percentages were calculated with missing observations so the total may not add up to 100.00.

**Figure 2:**
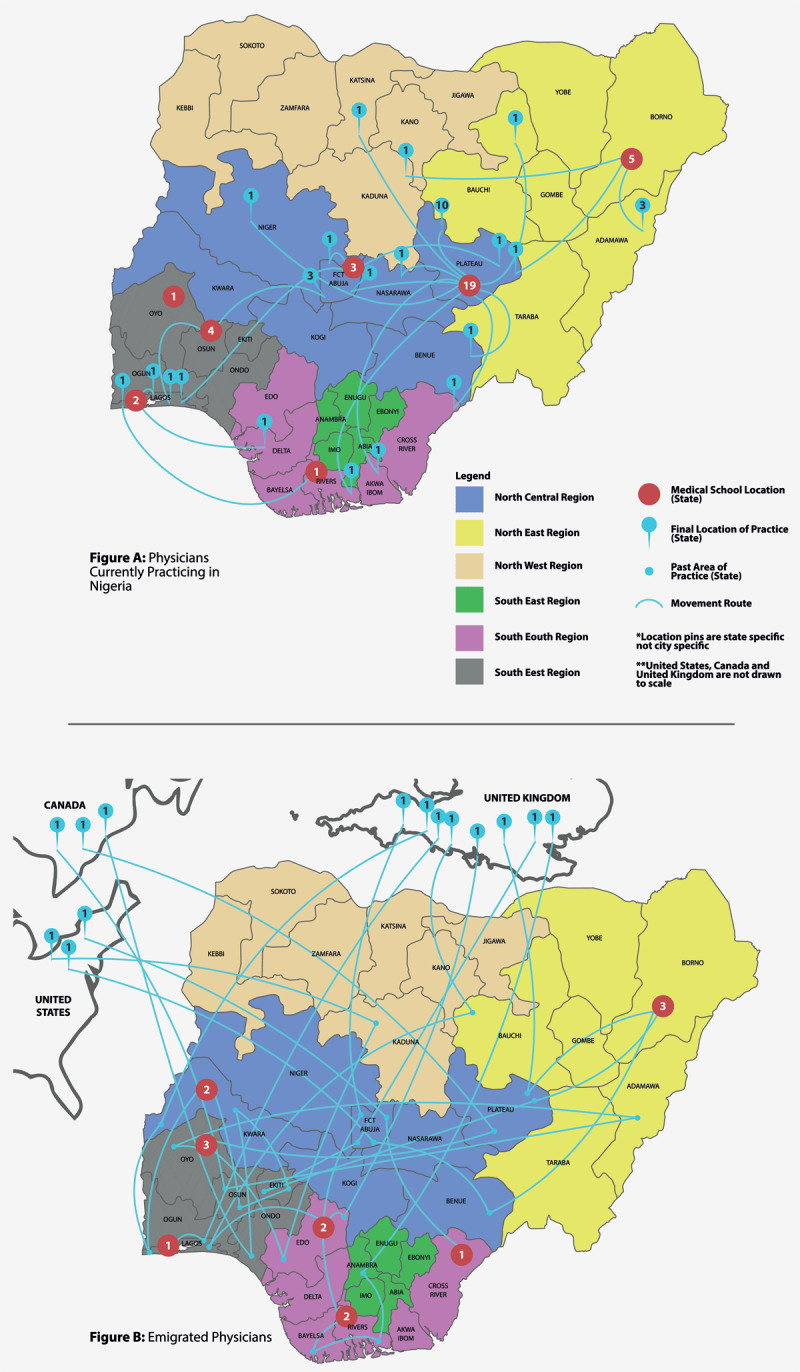
Pattern of physician movement from medical school to location of current practice(s).

The pattern of physician movement from medical school location, to location of current practice(s) are illustrated in Figure 2, (A) depicts the movement of physicians currently practicing in Nigeria, showing an overall flow of movement from the Northeast of the country—particularly Borno State where most of the *Boko Haram* related unrest currently is—to more Southern, Western and Middle-Belt regions; (B) depicts the movement of emigrated physicians, showing initial moves within Nigeria—which follow the same pattern of movement from regions of unrest— to final location of practice outside the country. The numbers of physicians per each state are represented in the location pins.

### Resilience building themes and framework

**Figure 3:**
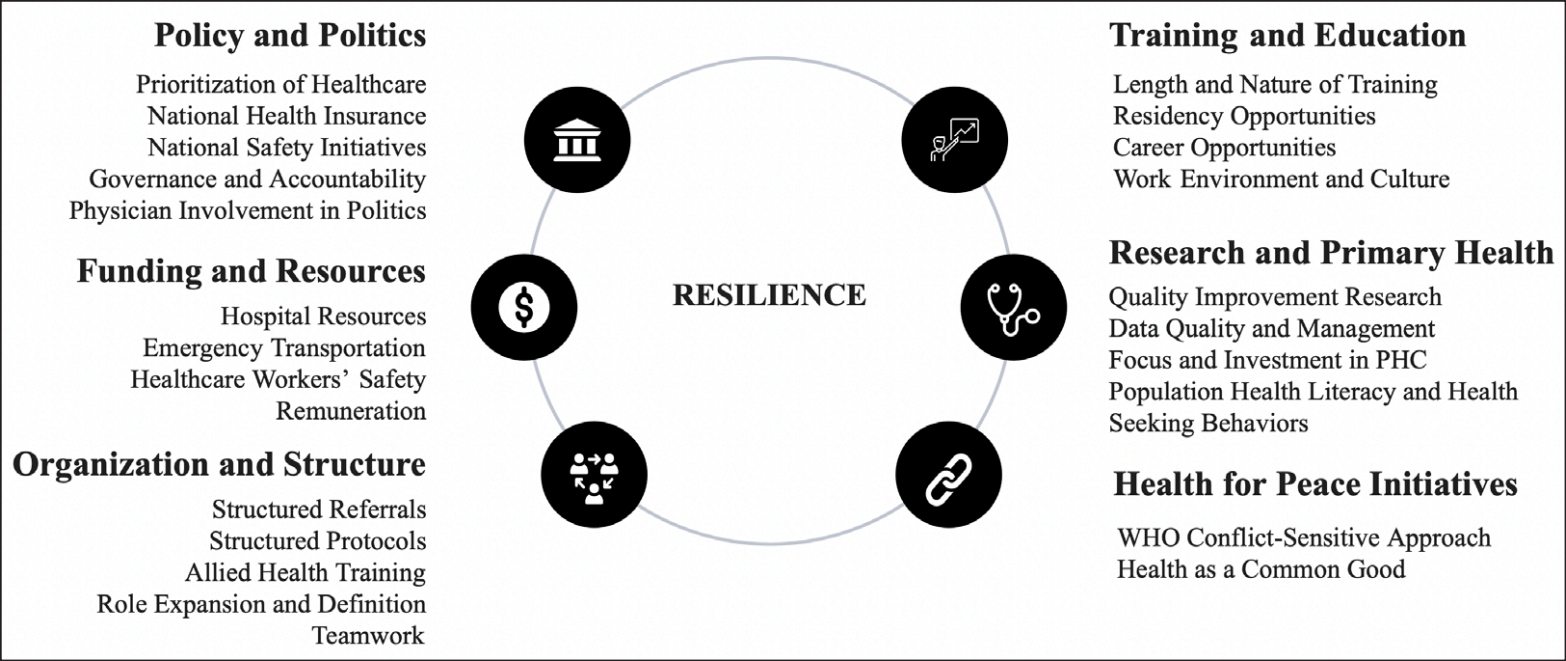
Resilience building themes and framework.

Figure 3 presents a resilience framework synthesized from the resilience building themes extracted from survey responses.

Suggestions from physician respondents on how the Nigerian healthcare system can maintain and promote resilience amidst the ongoing physician emigration and sociopolitical unrest fell into the following broad themes:

1. ***Policy and Politics***
  Regarding policy and politics, respondents drew attention to the effects of poor national security and the lack of healthcare prioritization on resilience building. One respondent commented that “the environment of Nigeria currently is such that it’s like a jungle. Anything hurtful can happen to you [at] any moment and you will find no help, even from the official law enforcement agencies, as they too are completely overwhelmed and generally compromised.” Another stated, “the fear of being kidnapped is rife every moment and the uncertainty of the security apparatus that exists makes daily living and going to work a risk in itself.” This has all led to a palpable emigration of workforce out of the country, as well as a move within the country—from the areas of highest insecurity and unrest. As one respondent noted, “unrest in the North means most doctors pool in certain areas (mostly Southern), and this further skews the doctor-patient ratio.”
  Thus, some of the suggested solutions for resilience building that target the shortcomings of policy and politics include the re-prioritization of healthcare and the formation and expansion of a national health insurance program which they hope will lead to widening access and decreasing gaps in care. Other suggestions include the creation, prioritization, and implementation of national safety initiatives with particular focus on “policies to ensure the safety of doctors and other allied health workers, especially at their places of work.” More accountability in governance and lack of complacency in political knowledge, as evidenced by physician involvement in politics, was also suggested, with one respondent remarking that, “doctors need to begin to forage into politics and mass advocacy to influence and lobby politicians with stolen wealth, and corner it into building and growing the health system.” However, some respondents were of the impression that, “there are good policies and guidelines [already in place], however they are not enforced or adhered to,” going further to suggest that “everyone needs to be lectured on these guidelines so that everyone knows what to do [and] how to do it.”
2. ***Funding and Resources***
  Responses point to the fact that funding and resources are drained by unrest, with one respondent noting, “funding that [could] be directed towards health care is directed towards security….” In addition, shortcomings in terms of policy and governance also emerged as reasons for the apparent lack of funds. Respondents believe that if funding is available for hospital resources, emergency transportation, healthcare workers’ safety and remuneration, resilience of the Nigerian healthcare system would be attainable. Some respondents suggest the, “review [of] costs of care and in turn employee remunerations to reflect the prevailing economic situation in the country [with use of] trade and business owners’ unions to create a united front so that standards can be uniform” and “partnership with more non-governmental organizations,” as innovative ways to tackle the funding deficit and channeling of funds to the healthcare sector.
3. ***Organization and Structure***
  Respondents also highlighted the current void of structured referrals and protocols, with suggestions around the, “standardization of practice with provision of guidelines for each area of practice.” In addition, suggestions highlighted creating a culture that fosters teamwork and responsibility, where roles are defined and considered as a team goal versus the current state described by one respondent as, “the constant back and forth with other healthcare workers for patient welfare, i.e., having to beg other people to do their jobs and many times needing to take on those responsibilities; [for example] doing nursing bedside procedures because the nurses wouldn’t do them.”
  Respondents also suggest the idea of creating opportunities for locums, especially by private sector health practitioners in government health facilities to address the workforce shortage, as well as to foster collaboration between sectors. Furthermore, respondents suggest a shift towards role expansion of allied health in the form of training opportunities like, “train[ing] Community Health Extension Workers (CHEWs)” to “build the capacities of non-doctors in the healthcare system, so they interface with patients at a primary level (with the use of checklists and algorithms) and escalate to the secondary/tertiary level where necessary.” Respondents believe that these will “improve workplace relations and reduce the burden on doctors,” thereby improving the resilience of the healthcare system.
4. ***Training and Education***
  Respondents noted the limited training and educational opportunities as well as the expenses associated with medical education as aggravating factors for emigration. Thus, the need for improvements in residency and career opportunities were posed, with one respondent suggesting, “structured residency training programs with obvious yearly evaluations” as a way to maintain and monitor training standards. Furthermore, suggestions to improve retention revolved around improving wellness amongst staff and addressing the hidden curriculum in the culture of medicine that could lead to “workplace toxicity, [where about] 7 in 10 Nigerian doctors are traumatized by bullying at the workplace [which] definitely hinders productivity and doesn’t allow one to fully learn how to or manage a lot of situations.”
5. ***Research and Primary Health***
  Respondents emphasized the deficits of quality improvement research and the primary health care (PHC) sector as a whole, suggesting ways to address these deficits by bolstering quality improvement research, funding to the primary health care (PHC) sector, population health literacy and health seeking behaviors. One respondent suggests, “research in the country to generate numbers and aid with health education” and another “[the] use of social media [to improve] patient education.”
  Some suggestions revolved around channeling more funding to research and data management with hopes to inspire quality improvement as a resilience building tactic, while others focused on the idea of, “expand[ing] the primary health care scheme and fuel[ing] it with surplus money, thereby creating more jobs for doctors.” As one respondent put it, “we can strengthen our primary healthcare system to build the capacities of non-doctors in the system”, with another remarking that “if the patient burden is slowly shifted to primary health care and preventive medicine, we could reduce the workload of the specialists, and in a way, address the poor doctor-patient ratios.” Furthermore, the theme of e-medicine, telehealth, and Electronic Medical Records (EMR) emerged as ways to help improve data quality, research, and patient health literacy.
6. ***Health for Peace Initiatives***
  Respondents also acknowledged the importance of peace in cultivating health. With the WHO’s conflict-sensitive approach as a means to create a starting point for bringing people together, the theme of, “health as a common good” emerged from the responses [15]. Some respondents mentioned that when health is viewed as a common good by the community at large, “no hospitals or health facilities [are] attacked during uprisings, ensuring adequate provisions of essential resources, [with] physicians outside the hospital ferried to and fro securely.” In addition, others highlighted the importance of a conflict-sensitive approach where health literacy is preserved, with one respondent stating that, “if the perpetrators of this unrest are members of the community, it’s possible to enlighten them on the importance of medical personnel in their communities.”

## Discussion

This study aimed to capture Nigeria-trained physicians’ perspectives regarding the ongoing physician emigration and sociopolitical unrest, the impact of the emigration and unrest on the local healthcare system, and key resilience building strategies for maintaining the healthcare system amidst these destabilizing factors. Utilizing a global health equity lens which is anti-oppressive and prioritizes capacity building, we employed an Asset-Based Community Development (ABCD) approach to community-based development which intentionally counteracts the deficit-oriented mentalities which perpetuate and reinforce colonial power dynamics [16,17,18]. Therefore, this study represents a response to the call to move decolonization of global health from reflection to action [18,19,20,21].

The resilience framework, which serves to shine light on key issues for consideration by health care policy planners whose aim is to stem mass physician migration, acknowledges that health system resilience is mediated by multiple and cumulative levels of adaptability with the involvement of multiple stakeholders [1,22,23,24,25,26,27]. Quantitative results with regards to plans for emigration and safety concerns indicate that the Nigerian healthcare system is not only currently unstable, but is also vulnerable to future instability in the form of continued progression of sociopolitical unrest and emigration. Therefore, resilience-building efforts should be distinguished from health system strengthening efforts; where the latter involves the health system planning for sudden instability and crises, resilience-building efforts should further include system planning that would also be sufficient to align resource deployment with *routine* healthcare needs in periods of stability [1,23,28,29,30,31,32].

The themes outlined in Figure 3 can serve as actionable points to tackle the tasks of resilience building, with steps that mirror the key components of the WHO health system framework—particularly health workforce, financing, and leadership and government—which form the building blocks of a health system [33]. Similarly, the WHO put out a toolkit for assessing health system capacity for crisis management, which includes a spate of declarations and agreements relating to strengthening health system capacity for disaster preparedness, further underlining the urgent need for all countries to be prepared to meet emerging threats to public health [34].

In particular, the emerging themes surrounding utilizing conflict-sensitive approaches to view health as a common good is reflective of the current challenges the Nigerian healthcare system is faced with. Specifically, the Northeast of the country has experienced attacks from the militant group known as *Boko Haram* since 2011; with the group initially targeting the police and churches, and then expanding to target mosques, schools, hospitals, and banks with bombings, military raids robberies and kidnappings [35]. These atrocities spread from the initial pressure point of Yobe State to the neighboring states of Borno and Adamawa, until a state of emergency was declared in these states in May 2013 [35]. This unrest has led to the internal displacement of inhabitants of the Northeast, as well as emigration from hardest hit regions, which also affects the healthcare workforce as highlighted in Figure 2 (A) above. Thus, these themes can be linked to the WHO Global Health for Peace Initiative (GHPI) which emphasizes using a conflict-sensitive approach to avoid doing harm, to increase project acceptance, and to mitigate risks, under the impression that “there cannot be health without peace, and there cannot be peace without health;” suggesting that health is viewed as a common good by all sides of a conflict, which allows health initiatives to serve as a starting point for bringing people together [15]. Nevertheless, there is more to be explored as to how this can be used effectively in Nigeria’s healthcare system amidst current strains.

The sense of urgency for this study is also palpable due to the potential for fragmented care and limited access—ultimately leading to poorer outcomes—that some argue is perpetuated by the current healthcare delivery model employed in Nigeria. Nigeria operates a pluralistic health care delivery system (orthodox and traditional health care delivery systems), with more focus on the orthodox or “Western” health care model whose care services are provided by private and public sectors [36]. However, the provision of health care in the country remains the functions of the three tiers of government (the federal, state, and local government)—with the primary health care (PHC) system managed by the local government areas (LGAs) with support from their respective state ministries of health as well as private medical practitioners; the secondary health care system managed by the ministry of health at the state level, and tertiary care provided by teaching hospitals and specialist hospitals (with the secondary and tertiary levels also working with voluntary and nongovernmental organizations, as well as private practitioners) [36,37]. Thus, the complexity of this model can exacerbate the issues surrounding care delivery and access in the current system.

As exposed in this study, physicians—who are major care delivery agents in the healthcare system—believe that the current model of healthcare delivery is greatly lacking in the PHC sector; highlighting gaps with regards to access and health insurance, which drive increases in out-of-pocket expenditure for healthcare. This can serve as a deterrent to the population actively participating in their healthcare, further hindering population health literacy, and leading to poor health seeking behaviors. This illuminates areas for future study and policy reforms which are consistent with the literature around the brain drain and the Nigerian healthcare system deficits [36]; further highlighting the importance of actively involving the healthcare workforce of LMICs—in this case, physicians—in seeking out innovative solutions for resilience building.

Furthermore, as a product of collaboration, this study highlights physicians trained in Nigeria as a relatively untapped resource in terms of resilience building, who possess a wealth of knowledge and are willing to participate in change. Therefore, regardless of the complexity of the task ahead, the results of this study can assist the stakeholders involved in health system resilience building by striving towards feasible solutions to instability amidst emigration and unrest.

### Limitations

This study has several limitations. First, the sample size is relatively small with only 49 participants included in the final analysis which may limit the conclusions we can draw from the study. However, despite a small sample size, the included participants were quite diverse in terms of their medical school background, practicing location(s), years of practice, and areas of practice. Second, the survey link was disseminated via *WhatsApp* with the ability for the group members to pass it on to their colleagues. Such convenient snowball sampling is prone to sampling bias. However, there is no database available to reach out to all Nigeria-trained physicians to conduct a randomized study; and the decision to refrain from collaborating with specific medical schools in Nigeria hinges on our goal to capture a wide range of physicians with diverse education and training within Nigeria. Furthermore, considering that the study is mostly qualitative, aiming to capture the challenges and suggestions around resilience building in the face of workforce emigration and unrest, we believe that the themes captured and discussed in the paper are justified. In the future, plans to increase participation by adding compensation and increasing the length of time the survey is available should be considered.

## Conclusions

The factors causing instability to healthcare systems globally are complex and diverse, with workforce emigration and sociopolitical unrest being two prominent factors of instability with solutions that are dynamic and variable. Nevertheless, this study suggests ways to maintain and promote resilience by utilizing the suggestions of locally trained physicians and synthesizing a framework for resilience for healthcare systems. The framework unearthed six broad themes: 1) Policy and Politics, 2) Funding and Resources, 3) Organization and Structure, 4) Training and Education, 5) Research and Primary Health, and 6) Health for Peace Initiatives. Some actionable points for resilience building which were developed as subordinate themes were also highlighted.

While the themes around the impact of physician emigration and sociopolitical unrest on the healthcare system are uniquely captured through the perspectives of Nigeria-trained physicians, these challenges—and the recommendations to mitigate them—are not unique to Nigeria’s healthcare system. Therefore, we believe the lessons learned through this study can be applied to other LMICs with similar challenges. Future studies should further develop how each of the six themes outlined here can be adapted with context-specific actionable points involving the participation of the local stakeholders.

## Data Availability

The survey datasets analyzed during the current study are available from the corresponding author on reasonable request.

## Abbreviations

ABCD–Asset-Based Community Development

EMR–Electronic Medical Record

GHPI–Global Health for Peace Initiative

HICs–High Income Countries

LGAs–Local Government Areas

LMICs–Low- and Middle-Income Countries

MDG–Millennium Development Goals

PHC–Primary Health Care

REB–Research Ethics Board

UN–United Nations

WHO–World Health Organization
